# Imaging Diagnosis of Hydrocephalus in a Fox Cub-Case Study

**DOI:** 10.3390/life15081159

**Published:** 2025-07-22

**Authors:** Alexandru Gabriel Neagu, Ruxandra Pavel, Ioana Ene, Raluca Mihaela Turbatu, Cristina Fernoaga, Niculae Tudor, Mihai Musteata

**Affiliations:** 1 Faculty of Veterinary Medicine, University of Agronomic Sciences and Veterinary Medicine of Bucharest, 011464 Bucharest, Romania; alexandru.neagu@fmvb.usamv.ro (A.G.N.); ioana.ene@fmvb.usamv.ro (I.E.); raluca.turbatu@fmvb.usamv.ro (R.M.T.); cristina.fernoaga@fmvb.usamv.ro (C.F.); 2 Faculty of Veterinary Medicine, University of Life Sciences “Ion Ionescu de la Brad”, 700490 Iasi, Romania; mihai.musteata@iuls.ro

**Keywords:** electroencephalography (EEG), fox, hydrocephalus, MRI

## Abstract

Hydrocephalus is a frequently observed congenital malformation of the central nervous system in domestic animals; however, its occurrence in wild species remains underreported. This study documents a clinical case of congenital hydrocephalus in a red fox cub (*Vulpes vulpes*) admitted to the “Visul Luanei” Wildlife Rehabilitation Center. The individual exhibited neurological deficits characterized by depressed mental status, incoordination, dromomania, behavior changes, and blindness. Diagnostic imaging, including radiography and magnetic resonance imaging (MRI), revealed a domed cranial morphology and severe dilation of the ventricular system. Notably, the lateral ventricles were markedly enlarged, with the absence of the septum pellucidum, resulting in a unified ventricular cavity. During electroencephalography (EEG) performed under general anesthesia, a high voltage and low frequency, predominantly featuring delta waves background activity was observed on all traces. Due to the poor prognosis and lack of clinical improvement, euthanasia was performed. This case contributes to the limited knowledge regarding central nervous system malformations in wild canids and underscores the need for further research on congenital neurological disorders in wildlife species.

## 1. Introduction

Hydrocephalus is a malformation of the ventricular system in which cerebrospinal fluid (CSF) accumulates in excess within the ventricular system as a result of either an increase in CSF production or a decrease in CSF absorption due to an underlying multifactorial disorder [1]. The increased accumulation of cerebrospinal fluid (CSF) raises intracranial pressure, leading to compressional atrophy and thinning of the surrounding brain parenchyma, and ultimately resulting in severe ventricular distention. It is associated with a wide variety of diseases and syndromes and can be congenital or acquired. Acquired hydrocephalus has been reported with numerous conditions, including obstruction, loss of brain parenchyma from infection with subsequent ventricular enlargement, or, in rare cases, increased CSF production from a choroid plexus tumor [2]. The etiopathogenesis of congenital hydrocephalus remains incompletely understood, particularly with respect to its genetic determinants. Although evidence of hereditary predisposition has been observed in certain domestic animal breeds, comprehensive genomic investigations remain scarce, limiting our understanding of the underlying molecular mechanisms [3].

The presence of wild terrestrial carnivores in urban and peri-urban sites has increased considerably over the years, implying an increased risk of interspecies spillover of infectious diseases and the transmission of zoonoses [4]. Foxes (*Vulpes vulpes*) are highly adaptable predators [5] and a specific interest is shown in the ethical control of the wild population of foxes [6]. This is because of their susceptibility and risk of developing zoonotic viral and parasitic encephalitis. Known causes of fox encephalitis include rabies, canine distemper, toxoplasmosis, Aujeszky’s disease, as well as parvovirus, adenovirus, circovirus, flavivirus, and birnavirus. In addition, foxes are known to be highly susceptible to infections of the order *Mononegavirales* [7,8]. Furthermore, the species is involved in several parasitic infestations with zoonotic implications [9,10,11,12], but also in antibiotic resistance [13].

Despite the risks presented above, there are a limited number of studies describing the clinical and paraclinical investigations (including advanced imaging or electroencephalography) in living foxes, especially juvenile ones with nervous system symptomatology. In a recent study on 79 foxes from which 15 were juvenile (trapped, hunted, or found dead), Lempp C. (2017) describes neurological lesions mostly represented by lymphohistiocytic perivascular encephalitis (11.4% of cases) and meningeal involvement in some other but no virus or parasitical etiological agent was identified [4]. In a study investigating the CT findings and histological evaluation of red foxes (*Vulpes vulpes*) with chronic head trauma injury, the authors found the presence of cranial vault fractures in all the included individuals associated with abnormalities of the brain parenchyma and hemorrhage, and in one case, the presence of hydrocephalus [14].

This study aims to provide the first EEG assessment and contribute to the scarce MRI data on congenital hydrocephalus in juvenile foxes and to present clinical, advanced imaging, and electrodiagnostic findings in a cub with forebrain symptomatology due to presumptive congenital hydrocephalus.

## 2. Case Description

A juvenile male fox cub (*Vulpes vulpes*) was rescued in Iasi County (eastern Romania) and temporarily cared for during the initial rescue phase, where it weighed approximately 800 g. After 3 to 4 weeks of supportive care, including assisted feeding and hydration, the cub was transferred to the “Visul Luanei” Wildlife Rehabilitation Center (WRC) in Bucharest, Romania, where it weighed approximately 1600 g upon arrival. Based on the eruption pattern of deciduous teeth and age-determination criteria specific to red foxes (*Vulpes vulpes*), the cub was estimated to be approximately 4 months old at the time of admission [15,16]. The body length was measured at 38 cm. Although these morphometric parameters differed slightly from the typical range reported for free-ranging cubs of similar age, they remained within the natural variation observed in wild populations.

Following admission, the cub was monitored in an intensive care unit for two weeks, during which it gained an additional 300 g. For comparison, other cubs of similar age at the same center typically weighed around 2.5 kg after a similar period of care. Throughout a three-week recovery period, persistent abnormal behaviors were documented—including difficulties in food acquisition and shelter-seeking, lack of foraging behavior, and absence of social integration within the group [17]. These clinical signs warranted referral to the Faculty of Veterinary Medicine in Bucharest—University Emergency Veterinary Hospital “Professor Dr. Alin Bîrțoiu”—for further neurological evaluation.

**Figure 1:**
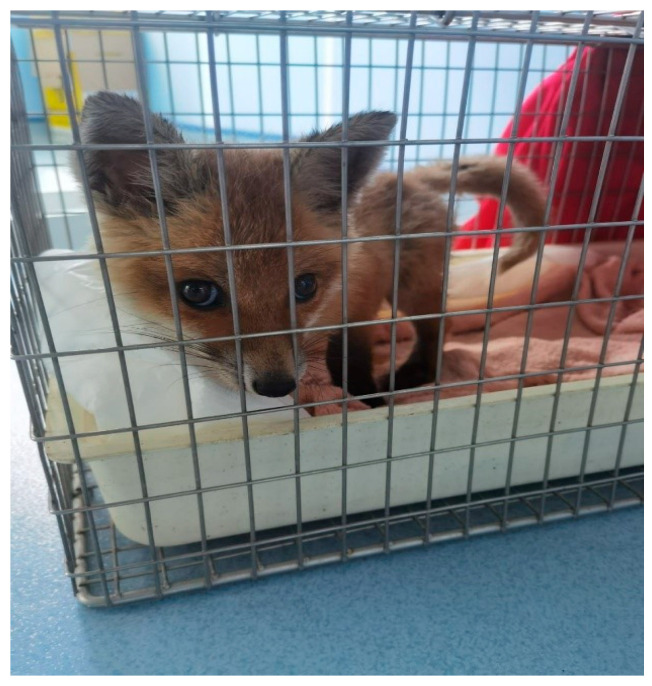
A 4-month-old male red fox (*Vulpes vulpes*).

At admission, the clinical evaluation revealed an underdeveloped patient, with cachexia and a 2 out of 5 condition score [18], with preference to adopt sterno-abdominal decubitus. The cub had a mildly enlarged circumference of the skull. The skull palpation revealed that the fontanelles were open. Neurological examination showed a depressed mental status and disoriented behavior. During gait, the patient manifested incoordination, dromomania, with the tendency to walk continuously on the side of the room. Sometimes he bumped into objects and appeared to be blind. Postural reactions were largely within normal limits, except for delayed responses observed during wheelbarrowing, extensor postural thrust, hopping, and hemiwalking tests. These deficits were bilaterally symmetrical, suggesting diffuse or central nervous system involvement rather than a focal lesion. Spinal reflexes were normal, and the patient was continent for both urine and feces. Cranial nerve examination revealed that the fox cub was clinically blind, as demonstrated by the absence of a menace response and failure to track a falling cotton ball, while bilateral direct and consensual pupillary light reflexes remained intact. A fundoscopic examination could not be performed due to the animal’s defensive and aggressive behavior during handling, which posed a significant safety risk. Olfactory function was evaluated by presenting a non-irritant, strong-smelling substance near the nares without direct contact, to specifically assess the olfactory nerve. In a separate step, light tactile stimulation of the nasal mucosa was performed using blunt-tipped forceps to evaluate local trigeminal sensitivity. Both olfactory response and local tactile reflex appeared mildly reduced bilaterally, and this distinction was made to avoid overlapping sensory modalities during cranial nerve assessment. In addition, a bilateral positional ventrolateral strabismus was noted (Figure 1), more pronounced in the right eye. Given the nature of this being a single-time examination and the presence of craniofacial malformations, it remains uncertain whether the strabismus was of neurological or developmental origin.

Based on the age, clinical and neurological findings of the cub and considering the non-progressive and symmetric evolution, a diffuse forebrain condition was suspected: as so, a posttraumatic lesion, anomaly, or a metabolic disturbance was privileged. However, an inflammatory/infectious etiology, a neoplastic, or a degenerative etiology was considered to be a differential diagnostics [19]. To establish the final diagnosis, additional investigations, including complete blood count, serum biochemistry, abdominal echography, radiological examination, MRI, and electroencephalography (EEG), were initiated.

The routine blood tests (hematology, serum biochemistry) and abdominal echography were within normal limits, so a special focus was made on the structural and functional evaluation of the skull and brain, using radiographies, MRI, and electroencephalography (EEG), was made. For these investigations, the animal was subjected to general anesthesia.

Patient preparation included a 3 h fasting with a water restriction of 30 min prior to premedication. Preanesthetic examination was performed, and the patient was assigned to ASA III status (American Society of Anesthesiology) [20]. Premedication was made with 1 mcg/kg dexmedetomidine (Dexdomitor^®^ 0.5 mg/mL Zoetis, Orion Corporation, Espoo, Finland), 0.2 mg/kg butorphanol (Butomidor^®^ 10 mg/mL, VetViva Richter GmbH, Wels, Austria), and 1 mg/kg ketamine (Ketamidor^®^ 100 mg/mL, VetViva Richter GmbH, Wels, Austria) given intramuscularly (IM). After premedication, a 26-G catheter was placed in the cephalic vein. Induction was made with 2 mg/kg propofol (Fresofol^®^ 1% MCT/LCT, Fresenius Kabi Australia, Mount Kuring-gai, Australia) intravenously (IV). The patient was intubated with a 4 mm endotracheal tube, and maintenance of anesthesia was performed with isoflurane (Isothesia, 1000 mg/g, Piramal Critical Care B.V., Voorschoten, The Netherlands) in 100% oxygen. Both eyes received 2 drops of a lubricant ophthalmic gel with 1% hyaluronic acid for cornea protection [21]. Oxygen flow was initially delivered at 2 L/min, 2.0% isoflurane within 3–5 min of induction. After 3–5 min, oxygen flow decreased to (500 mL + 10 mL/kg) per minute, and isoflurane was maintained to a mean minimum alveolar concentration (MAC) of 1.7. The patient breathed spontaneously under anesthesia. Lactated Ringer solution (B. Braun Medical S.R.L. 500 mL, Timiș, Romania) was administered IV at 4 mL/kg/h throughout anesthesia. During MRI, the patient had a silicone hot water bottle (35 cm × 20 cm) placed under the abdomen, with the entire body also covered by a polyester cloth blanket to prevent hypothermia [22]. No complications were recorded.

**Figure 2:**
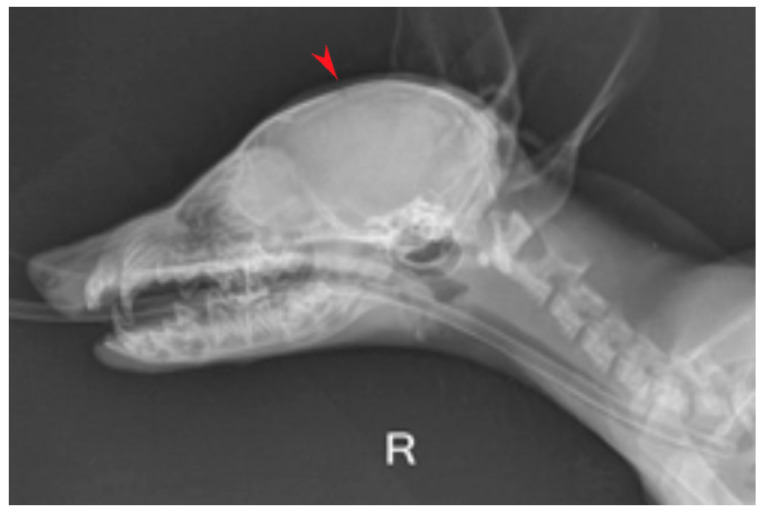
Right latero-lateral view, domed shape calvarium and open fontanelles (red arrow).

For the imaging examination, a direct digital radiology machine (Dura Diagnost F30, Philips Healthcare, Suzhou, China) was used: the skull bones and soft tissue were examined through two views (right latero-lateral and dorso-ventral) for highlighting changes at this level. The radiological examination showed an abnormally enlarged skull with a dome-shaped calvarium and open fontanelles (Figure 2).

**Figure 3:**
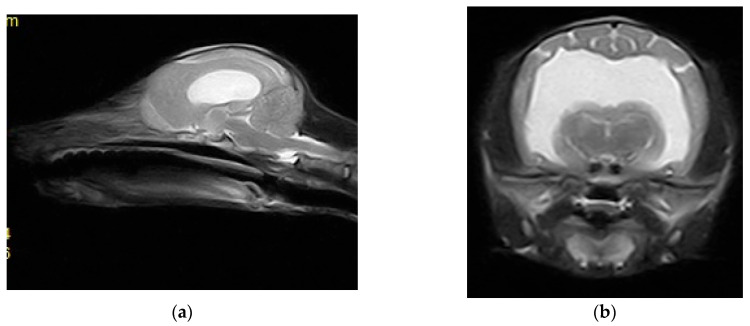
(**a**) Sagittal T2 sequence showing enlargement of the ventricular system and loss of the pellucid septum; (**b**) transversal T2 sequence showing the severe enlargement of the lateral ventricles, with loss of the pellucid septum and a marked communication between them.

**Figure 4:**
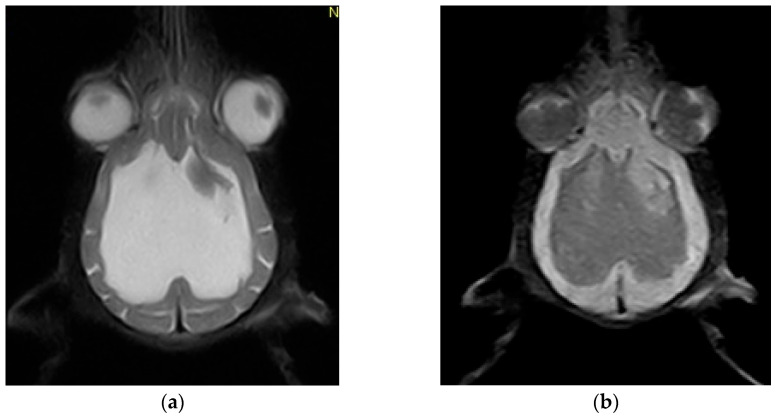
(**a**) Dorsal T2-weighted sequence showing marked thinning of the cerebral cortices, enlargement of the lateral ventricles, and absence of the septum pellucidum; (**b**) Dorsal FLAIR (Fluid-Attenuated Inversion Recovery) sequence confirming the ventricular dilation and cortical atrophy. Notably, the apparent asymmetry in the ventricular system is not genuine but rather attributable to a mild artifact.

Magnetic resonance imaging (MRI) scans were acquired using a low-field 0.3 Tesla system (Vet-MR Grande, Esaote, Italy) with the dedicated head coil. To highlight the changes for the study, a complete MRI series was taken, including sagittal, transverse, and dorsal T2-weighted (T2W) images, transverse fluid-attenuated inversion recovery (FLAIR), as well as transverse pre-contrast and post-contrast T1-weighted (T1W) images, with thickness between 3 and 3.5 mm at the brain level. MRI shows CSF liquid hyperintensity signal on T2W and hypointensity signal on T1 and confirmed by FLAIR images which shows a severe ventricular enlargement with thinning of the cerebral cortices, absence of the pellucid septum that leads to continuity between the lateral ventricles (marked communication), and no dilatation of the olfactory recesses was seen (Figure 3 and Figure 4). Based on the MRI findings, a presumptive diagnosis of congenital hydrocephalus was suspected.

**Figure 5:**
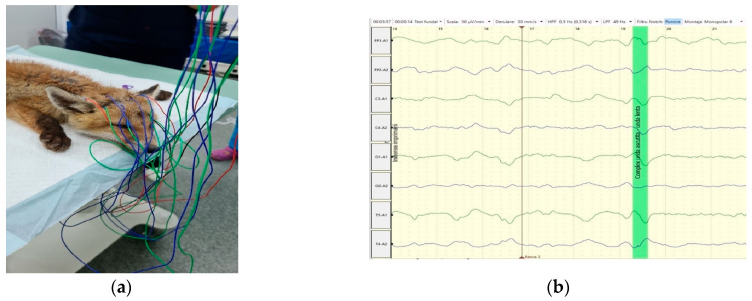
(**a**) EEG recording using monopolar electrode placement, with ipsilateral ear referential montage, in a red fox (*Vulpes vulpes*); (**b**) multiple EEG waves, with no pathological grapho-elements.

After the MRI investigation, a 20-minute EEG recording was made using a Neuron-Spectrum 4/P EEG machine (Neurosoft, Ivanovo, Russia) and a montage of eight subdermal stainless-steel needle electrodes (F1, F2, C3, C4, T3, T4, O1, O2) in both referential and bipolar montage (Figure 5). Recording parameters were: impedance < 5 kΩ, sensitivity = 50 μV/cm; time constant = 0.3 s; Hf = 70 Hz; Lf = 0.5 Hz; notch filter inserted. The reference electrode was placed on the ear, and the ground on the neck.

The visual analysis of the EEG revealed a background activity (BGA) marked by high voltage and low frequency, predominantly featuring delta waves [23]. No pathological grapho-elements (spike, polyspikes, spike-waves, etc.) were identified on recorded traces.

Based on clinical data, neurological signs, imaging diagnosis, and EEG interpretation, a diagnosis of presumed congenital hydrocephalus was established.

While congenital hydrocephalus is a relatively common condition observed in fox cubs [5,13,24,25], this case is distinguished by the incorporation of electroencephalographic (EEG) evaluation that provides functional data not previously documented in this species and contributes to a deeper understanding of its neurophysiological profile. In those animals, the condition is believed to result from infection with the *Ljungan* [26], but a genetic etiology cannot be excluded [24]. Neurological symptoms in dogs with hydrocephalus typically indicate the forebrain involvement and may imply abnormal behavior, lethargy, blindness, circling, and seizures [25]. Although the connection between seizures and hydrocephalus has been questioned in recent studies [27], convulsions are frequently reported in all the affected species, including foxes, in which a congenital form of hydrocephalus is relatively common [28]. In our case, the cub expressed clinical signs specific to a forebrain location, but no seizures were observed during the hospitalization period (over a month). In cubs, the altered mentation represents a life-threatening sign, as long as affected animals are usually abandoned by the vixen [28] and their ability to survive alone is limited. This is probably the main reason a very limited number of fox cubs with congenital hydrocephalus have been reported.

In any wild animal, a modified behavior should raise the question of a potential infectious encephalitis, and the risk of rabies should never be excluded. After the implementation of the EU foxes vaccination program in 2007, the number of animals reported in Romania decreased significantly, and positive cases are sporadically reported [29,30]. In our case, although rabies virus infection was not excluded from our differential, the static appearance of symptoms and specific structural changes (focal T2 hyperintensity) led to the exclusion of this diagnosis. Moreover, during over a month of hospitalization, the cub showed no improvement, and euthanasia was performed due to the bad prognosis.

Electroencephalography (EEG) has been validated as a reliable electrodiagnostic modality for evaluating the functional impact of hydrocephalus on cerebral electrical activity in dogs [31,32]. In the study by Armasu et al. [32], EEG abnormalities were documented in all six juvenile dogs (6/6) diagnosed with congenital hydrocephalus, indicating a consistent alteration in bioelectrical brain patterns. These alterations were characterized by markedly increased amplitude and low-frequency waveforms. Furthermore, the authors reported the presence of interictal epileptiform discharges—paroxysmal grapho-elements—suggesting underlying cortical hyperexcitability in addition to the modified background activity (BGA). Similarly, Brüssau et al. [33] described comparable EEG features, including high-amplitude, low-frequency activity, and variable epileptiform patterns, across their cohort of six hydrocephalic dogs. This EEG profile corresponds closely to the findings in the present case of the fox (*Vulpes* spp.) with congenital hydrocephalus, whose EEG trace also revealed high-amplitude, low-frequency wave activity. However, it must be noted that the EEG was recorded under general anesthesia, which may have influenced the observed patterns. In a vigilant (awake) state, the background activity may have included predominantly beta waveforms—thus, a different BGA pattern cannot be excluded. For the presented fox, we performed a short-time EEG. Therefore, we cannot exclude the occurrence of interictal epileptic discharges in longer EEG recordings or during the vigil state, so the coexistence of subclinical epileptiform activity cannot be excluded. However, during the hospitalization period, no epileptic attack was documented.

To the best of our knowledge, at present, there is no report regarding the brain bioelectric activity assessed by the EEG technique in a juvenile fox diagnosed with congenital hydrocephalus. Moreover, our results are expanding the limited number of reports regarding the MRI findings in congenital hydrocephalus in cubs.

We acknowledge that the absence of cerebrospinal fluid (CSF) analysis and histopathological examination represents some limitation of this study, as these investigations would have been critical for confirming or excluding inflammatory or infectious etiologies such as encephalitis. While CSF analysis is a valuable diagnostic tool, in cases of hydrocephalus, it carries a substantial risk of inducing brain herniation, particularly in small or unstable patients, and was therefore not performed ante-mortem. Furthermore, the fox cub displayed a static neurological condition over the course of approximately one month of hospitalization, an interval during which clinical deterioration or episodic exacerbation would typically be expected in immune-mediated or other chronic progressive conditions. In the context of this stable clinical course and supporting imaging findings, a congenital, non-inflammatory etiology was considered most likely, though this remains a presumptive conclusion in the absence of definitive post-mortem diagnostics.

## 3. Conclusions

Congenital hydrocephalus, although infrequently reported in fox cubs, may be underdiagnosed due to early mortality and maternal abandonment. In this paper, we present the clinical signs, as well as the MRI and EEG findings obtained under general anesthesia, in a juvenile fox with presumed congenital hydrocephalus. Although rarely reported, hydrocephalus should always be included in the differential in any cub with forebrain signs. MRI findings are similar to those observed in dogs, and the EEG revealed a high-voltage, low-frequency background activity dominated by delta waves, aligned with patterns previously described in juvenile dogs affected by congenital hydrocephalus. This case highlights the importance of timely diagnostic assessment, careful clinical monitoring, and emphasizes the clinical aspects of integrating neurological examination, advanced imaging, and EEG assessment in the diagnostic workup of wild animals.

## Data Availability

The data generated in this study are presented in this article. For any further information, the reader can contact the authors.
